# Truncated Active Human Matrix Metalloproteinase-8 Delivered by a Chimeric Adenovirus-Hepatitis B Virus Vector Ameliorates Rat Liver Cirrhosis

**DOI:** 10.1371/journal.pone.0053392

**Published:** 2013-01-03

**Authors:** Jinxia Liu, Xin Cheng, Zhengrong Guo, Zihua Wang, Dong Li, Fubiao Kang, Haijun Li, Baosheng Li, Zhichen Cao, Michael Nassal, Dianxing Sun

**Affiliations:** 1 The Liver Diseases Diagnosis and Treatment Center of PLA, Bethune International Peace Hospital, Shijiazhuang, People’s Republic of China; 2 Department of Traditional Chinese Medicine and Liver Disease, The Third Affiliated Hospital of Hebei Medical University, Shijiazhuang, People’s Republic of China; 3 University Hospital Freiburg, Internal Medicine II/Molecular Biology, Freiburg, Germany; Drexel University College of Medicine, United States of America

## Abstract

**Background:**

Liver cirrhosis is a potentially life-threatening disease caused by progressive displacement of functional hepatocytes by fibrous tissue. The underlying fibrosis is often driven by chronic infection with hepatitis B virus (HBV). Matrix metalloproteinases including MMP-8 are crucial for excess collagen degradation. In a rat model of liver cirrhosis, MMP-8 delivery by an adenovirus (Ad) vector achieved significant amelioration of fibrosis but application of Ad vectors in humans is subject to various issues, including a lack of intrinsic liver specificity.

**Methods:**

HBV is highly liver-specific and its principal suitability as liver-specific gene transfer vector is established. HBV vectors have a limited insertion capacity and are replication-defective. Conversely, in an HBV infected cell vector replication may be rescued *in trans* by the resident virus, allowing conditional vector amplification and spreading. Capitalizing on a resident pathogen to help in its elimination and/or in treating its pathogenic consequences would provide a novel strategy. However, resident HBV may also reduce susceptibility to HBV vector superinfection. Thus a size-compatible truncated MMP-8 (tMMP8) gene was cloned into an HBV vector which was then used to generate a chimeric Ad-HBV shuttle vector that is not subject to superinfection exclusion. Rats with thioacetamide-induced liver cirrhosis were injected with the chimera to evaluate therapeutic efficacy.

**Results:**

Our data demonstrate that infectious HBV vector particles can be obtained via *trans*-complementation by wild-type virus, and that the tMMP8 HBV vector can efficiently be shuttled by an Ad vector into cirrhotic rat livers. There it exerted a comparable beneficial effect on fibrosis and hepatocyte proliferation markers as a conventional full-length MMP-8Ad vector.

**Conclusions:**

Though the rat cirrhosis model does not allow assessing *in vivo* HBV vector amplification these results advocate the further development of Ad-HBV vectors for liver-specific gene therapy, including and perhaps particularly for HBV-related disease.

## Introduction

Liver cirrhosis is a chronic, progressive condition characterized by fibrosis and the conversion of normal liver architecture into structurally abnormal nodules [1], [2]. Frequently, liver cirrhosis precedes hepatocellular carcinoma [3]. Prevention, detection and therapy pose serious challenges to clinical management and engender major health costs worldwide [4], [5]. The etiologies of liver injury resulting in cirrhosis are diverse, but globally 30%, and in China up to 66% of cirrhosis are attributed to hepatitis B virus (HBV) infection [6]. Cirrhosis emerges successively from liver fibrosis [7], a progressive imbalance between fibrogenesis and fibrolysis leading to deposition of extracellular matrix (ECM) proteins, mostly from activated hepatic stellate cells [8]. Type I collagen accounts for 60%–70% of the total collagen in fibrotic livers [9], [10], [11].

Progression of experimental liver cirrhosis is accompanied by a gradual loss of collagenolytic activity in liver tissue [12], [13]. The major collagenases are the matrix metalloproteinases (MMPs) MMP-1, MMP-8, MMP-13 [14], [15]. They are synthesized as about 470 amino acid latent preproproteins which are activated by removal of the prepro sequences comprising the first 100 residues. Activation of MMPs is controlled by the tissue inhibitors of metalloproteinases (TIMPs) [16], [17]. Failure to degrade excessive ECM can thus result from insufficient expression and/or activation of MMPs, or from up-regulation of TIMPs. Plausible therapeutic approaches beyond removal of the fibrotic stimuli underlying liver injury [7] might hence include inhibition of TIMPs or increased expression of collagenases [7], [18], [19].

Experimental proof of concept for the latter strategy has been obtained in two studies that employed adenovirus (Ad) vector-mediated delivery of MMP-1 and MMP-8 genes into experimental rat models of fibrosis [20]. The results showed indeed regression of fibrosis and, as an additional benefit, induction of hepatocyte proliferation [21], [22].

**Figure 1:**
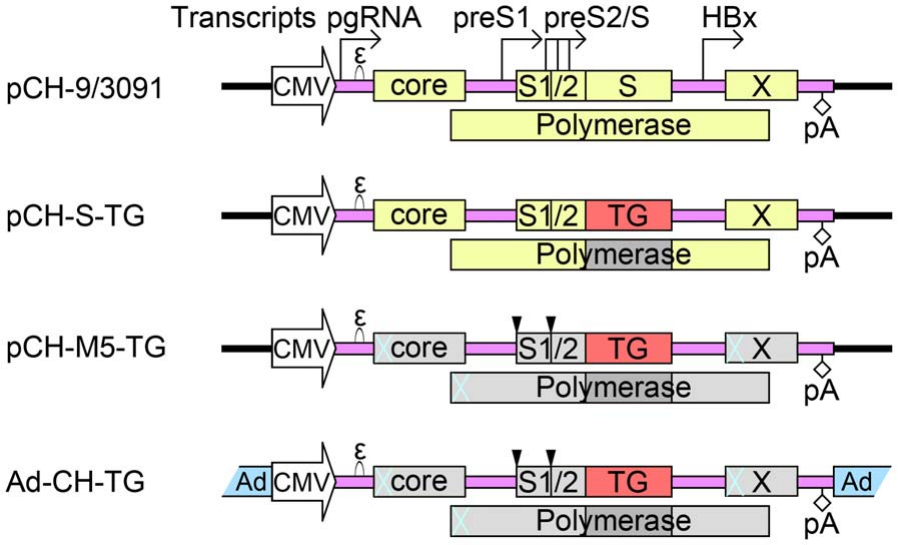
HBV vector constructs used. All constructs were based on the wild-type HBV expression plasmid pCH-9/3091 which contains a complete HBV genome. Open reading frames are depicted as yellow boxes. Transcription of the pregenomic (pg) RNA is under control of the CMV-IE promoter. The start sites for transcripts controlled by the endogenous preS1, preS2/S and HBx promoters are indicated by the rightward pointing arrows. “ε” denotes the RNA packaging signal whose interaction with the polymerase initiates pgRNA encapsidation and reverse transcription. The diamond labeled pA symbolizes the HBV polyadenylation signal. In first generation HBV vectors, represented by pCH-S-TG, the transgene (TG) replaced the viral S gene. Here, eGFP, RFP2 and Luc were used as transgenes. These vectors still produce core and X protein, plus truncated forms of polymerase (region covered with dark slash lines) and PreS1/PreS2 (pS1, pS2) gene products. For vector production, functional polymerase and surface proteins are provided *in trans* by a helper plasmid, e.g. pCH3142 which is identical to pCH-9/3091 except it lacks the “ε” signal. In the pCH-M5-TG vectors, expression of the endogenous HBV gene products was ablated by premature stop codons (blue crosses), or mutation of the preS1 and preS2 initiation codons (downward pointing triangles) so as to engage all preS/S transcripts as TG mRNAs; here TG included truncated MMP-8 (tMMP8). For the chimeric Ad-HBV vectors, Ad-CH-TG, the entire HBV vector expression cassettes from the pCH-M5-TG plasmids were incorporated into ΔE1/ΔE3 Ad-vector backbones. Ad-C-MMP8 contained full-length MMP-8 under CMV promoter control, but no HBV sequences.

Hence MMP delivery as such appears promising for treatment of liver fibrosis and cirrhosis yet application of Ad vectors in human liver gene therapy is currently restricted by issues of safety, and efficacy and sustainability of heterologous gene transduction [23]. At high doses favoring efficient transduction, the Ad vector particles may trigger excessive innate and adaptive immune responses. Less vigorous responses upon lower dose administration may still prevent repeated application of the vector although this may often be desirable due to the transient expression of Ad vector-transduced genes. Intraveneously administered Ad vectors accumulate preferentially in the liver [24], but they have an intrinsically broad tropism for epithelial-derived cells in other tissues [25], especially the upper respiratory tract, which may increase the risk of systemic delivery [23]. In contrast, HBV has a strict hepatocyte tropism making it a principally useful vehicle for liver-specific gene transfer. Moreover, transgenes controlled by the endogenous HBV promoter/enhancer elements may preferentially be expressed in hepatocytes, thus contributing to liver specificity. HBV and its related animal viruses, e.g. duck HBV (DHBV), are small (genome size about 3 kb) enveloped DNA viruses that replicate through reverse transcription [26], [27]. This requires that the greater-than-genome length pregenomic (pg) RNA be co-packaged with the viral polymerase into newly forming nucleocapsids, inside of which reverse transcription occurs. This and the extremely compact genome organization (see Fig. 1) with numerous overlapping open reading frames (ORFs) and regulatory *cis*-elements has hampered early attempts to harness HBV into a gene-transfer vector by simple insertion of foreign sequences. However, replacement of the S gene by similarly sized heterologous sequences turned out as a viable strategy [28]. Once the vector has entered the target cell and the incoming relaxed-circular (RC) DNA is converted into nuclear covalently closed circular DNA (cccDNA), transcription of the foreign gene is then controlled by the endogenous HBV preS1 and preS2/S promoters which in the authentic virus direct synthesis of the mRNAs for the L protein (PreS1/PreS2/S), and the M (PreS2/S) and S proteins (Fig. 1); especially the preS1 promoter shows a high specificity for differentiated hepatocyte-derived cells [29]. The simultaneous loss of functional surface proteins and polymerase is rescued by cotransfection of the vector with a helper plasmid such as pCH3142, which harbors a slightly truncated HBV genome that provides all necessary viral proteins *in trans* but itself is not packaged because the 5′ proximal RNA encapsidation signal ε is deleted [30]. HBV vectors obtained in this way selectively accumulated in the liver after inoculation into peripheral vessels, efficiently infected quiescent hepatocytes, and successfully transduced genes for green fluorescent protein (GFP) and type I interferon (IFN-α). The IFN-α vector substantially suppressed replication of resident HBV. These data suggested that HBV-based vectors may also become useful against other liver diseases [31]. However, appropriate effector genes have to be smaller than about 1 kb.

HBV vectors produced in this way are infectious but replication-defective, i.e. they cannot reproduce and transgene expression is transient. However, analogous to *trans*-complementation by a helper plasmid during vector production, a vector entering a wild-type HBV-infected hepatocyte may be rescued *in trans*, leading to a second generation of recombinant virions that can infect surrounding hepatocytes. Hence the therapeutic vector might be amplified *in situ* and maintain transgene expression as long as wild-type HBV is present. The most obvious application would be vectors that directly target the resident wild-type HBV because vector spread would be self-limited by the progressive loss of HBV infected cells capable of trans-complementation. The potential helper functions of wild-type HBV might also be capitalized upon when targeting the sequelae of chronic HBV infection such as liver cirrhosis, especially in the light of the large number of patients suffering from this condition.

At present, however, this concept is theoretical because several basic issues have not yet been experimentally addressed. Particularly important are the unknown ability of the HBV vector to be complemented by wild-type HBV rather than a designed helper construct, and overcoming superinfection exclusion by which a resident virus impairs infection of its host cell by another virus using the same infection route. For DHBV, this phenomenon is well established [32]. Not the least, therapeutic benefit of an HBV vector-based therapy has not yet been demonstrated in an *in vivo* disease model.

The aim of this study was therefore to address several of these outstanding questions, using the *in vivo* fibrosis model data available for full-length MMP-8 transduction by a conventional Ad vector [33] as a reference.

Several prior studies facilitated in realization of the current approach. First, previous data on HBV vector design [28], [34] provided a basis for further vector improvements. Second, a truncated MMP-8 encompassing aa 100–262, a-tMMP-8 (for simplicity termed tMMP8 below) can be recombinantly expressed and secreted and is still capable of degrading exogenously accumulated extracellular type I collagen [35]. Lacking the prosequence it does not require proteolytic activation, and with about 600 bp the coding region easily accomodates into HBV vectors. Third, we and others have previously demonstrated that complete wild-type HBV genomes can be incorporated into conventional Ad vectors and efficiently be transduced into human cell lines and primary hepatocytes, with production of HBV cccDNA, and into animals [36], [37]. This might also hold for recombinant HBV vector genomes. The different infection route of adenovirus vs. HBV should circumvent any superinfection exclusion problems, enabling efficient initial deposition of the Ad vector-shuttled HBV vector also in HBV infected cells. The results shown below strongly suggest that the concept of Ad-vector shuttled HBV vector delivery into the liver is feasible. Importantly, the truncated tMMP8 embedded into the HBV part of the chimeric Ad-HBV vector maintained key therapeutic features previously seen with conventional Ad vector delivery of full-length MMP-8 [33], [38], namely a similarly efficient reduction of fibrosis and cirrhosis, and in addition the activation of signals promoting hepatocyte proliferation.

## Materials and Methods

### Ethics Statement

This study was performed in strict accordance with the recommendations in the guidelines (Animal Research: Reporting *In vivo* Experiments) developed by the National Center for the Replacement, Refinement and Reduction of Animal’s in Research, London, UK. The protocols for animal experiments were approved by the Medical Ethics Committee of Bethune International Peace Hospital (Permit Number: 2009–19). Only the minimal number of required animals was used. All rats were kept under species-appropriate conditions, with sufficient supply and convenient access of food and water. All surgery was performed under sodium pentobarbital anesthesia, and every effort was made to minimize suffering.

### Cell Culture, Transfection, and HepaRG Cell Infection

HepG2 cells (American Type Culture Collection) were cultured in high glucose Dulbecco’s minimal essential medium (Thermo Scientific) supplemented with 10% heat-inactivated fetal bovine serum (Thermo Scientific), 2 mM L-glutamine, 100 µg streptomycin/ml, and 100 U of penicillin G ml^−1^ and buffered with sodium bicarbonate (complete medium), 1% (v/v) non-essential amino acid (Invitrogen), in a fully humidified atmosphere containing 5% CO_2_, 95% air at 37°C. Fugene HD (Roche) was used for plasmid transfection. HepaRG cells (Biopredic International, France) were cultured and differentiated as described [39]. For infection, viral particles in the culture supernatants from HepG2 cells cotransfected with a 1∶1 mixture of plasmids pCH-M5-GFP and pCH-9/3091 were concentrated by PEG 8000 precipitation. Prior to precipitation, DNase I (Takara) and micrococcal nuclease (Fermentas) were used to minimize residual plasmid DNA so as to exclude uptake and potential gene expression from internalized plasmids. Nominal viral titers, reflecting the sum of wild-type and recombinant virus, were determined by quantitative PCR (qPCR) with a commercial HBV DNA quantification kit (Shanghai Kehua Bio-engineering Co., Ltd, Shanghai, China) and expressed as viral genome equivalents (vge) per ml. Inoculation of the HepaRG cells was performed in the presence of 4% PEG 8000 as described [39], using about 5×10^8^ vge per 10^5^ cells in one well of a six-well plate. The virus inoculum was left on the cells for 48 hours and then replaced by fresh medium.

### Fluorescence Imaging and Luciferase Activity Assay

GFP expression was detected using a fluorescence microscope (Leica, Germany), 48 hours after transfection of HepG2 cells, or six days after infection of HepaRG cells. Expression of red fluorescent protein 2 (RFP2) in frozen sections of recombinant adenovirus infected rat liver was also detected by fluorescence microscopy. Firefly luciferase (pGL3-control, Promega) and Renilla luciferase activity were measured 48 hours after co-transfection by the Dual-Luciferase Reporter Assay System (Promega, USA) as recommended by the manufacture.

### Plasmid Construction

Plasmid pCH-S-GFP had been derived from the wild-type HBV (Genbank accession no: V01460, formerly J02203) expression vector pCH-9/3091 [40] by replacing the S gene with a gene of eGFP [28]. To prevent expression of endogenous HBV proteins, the following mutations were introduced into pCH-S-GFP, yielding plasmid pCH-M5-GFP (nucleotide numbers refer to the position of the A in core gene initiation codon): G28T (GGA to TGA), T444A (TTG to TAG), C2677T (CAA to TAA). These mutations introduce premature stop codons in the core, polymerase, and X gene respectively. Knock-out of the PreS1 and PreS2 start codons involved mutations T949C (ATG to ACG) and T1273C (ATG to ACG) [41]. Plasmids pCH-S-RFP2 and pCH-M5-RFP2, and the corresponding firefly luciferase constructs pCH-S-Luc and pCH-M5-Luc were obtained by appropriate gene substitution in pCH-S-GFP and pCH-M5-GFP, respectively. Vector pCH-M5-tMMP8 was analogously constructed by introduction of the PCR product obtained using the primers tMMP8Nco(+): 5′GGC GCC ATG GTA ACC CCA GGA AAC C and tMMP8Spe(−): 5′GCC GAC TAG TCA TCC ATA GAT GGC CTG AAT G on plasmid pGW1GH-MMP8 encoding full-length MMP-8 (Genbank accession no. NP_002415.1; kindly provided by Professor Christopher M. Overall, Canada) as template [42].

For Ad vector construction, the NheI fragments from pCH-M5-tMMP8 and pCH-M5-RFP2 comprising the CMV promoter plus the complete HBV vector part were transferred into plasmid pDC312-hrGFP, a derivative of the shuttle plasmid pDC312 (AdMax system; Microbix, Canada), yielding plasmids pDC-CH-tMMP8 and pDC-CH-RFP2. The corresponding shuttle plasmid for full-length MMP-8, pDC-C-MMP8, was obtained by subcloning the Spe I to BamH I fragment from pGW1GH-MMP8, comprising CMV promoter, full-length MMP8 coding sequence and SV40 polyA signal, into pDC312-hrGFP.

### Recombinant Ad-vectors and Ad-HBV Chimeric Vectors

Shuttle plasmids pDC-CH-tMMP8, pDC-C-MMP8 and pDC-CH-RFP2 were recombined with adenovirus rescue plasmid pBHGloxΔ-E1,3Cre in HEK293 cells according to the protocols provided with the AdMax system. Briefly, HEK293 cells were seeded in 6-well plates (1.2×10^6^ cells per well) 24 h ahead of transfection and cultured with 10% (v/v) fetal bovine serum supplemented DMEM (Invitrogen,USA) at 37°C, 5% CO_2_. Cells were then co-transfected with 5 µg shuttle plasmid and 25 µg rescue plasmid using FuGene 6 reagent (Roche). When visible plaques formed 15 to 20 days post transfection, cells and medium were harvested for virus preparation as previously described [36]. These viruses were then used to infect HEK293 cells in thirty 150 mm dishes. When more than 90% of the cells showed complete cytopathic effect (48–72 h later), viral particles were released from the pelleted cells by three freeze/thaw cycles and purified using cesium chloride density gradients. After removing CsCl from the viral stocks using NAP-25 columns (Sephadex G-25 Medium, Pharmacia), physical titers were determined via optical absorbance at 260 nm. The concentration was calculated using the formula1 OD_260_ unit  = 1.1×10^12^ virus particles/ml.

### Southern Blotting of HBV Constructs in Cells Transfected by Recombinant HBV

Southern blotting was performed as previously described [43], [44], [45]. Briefly, cytoplasmic extracts were obtained by suspending the cells in lysis buffer containing 10 mM Tris-HCl (pH8.0), 1 mM EDTA (pH8.0), 150 mM NaCl, 0.2% NP-40 and removing nuclei and cellular debris by centrifugation for 2 min at 13000 rpm, 4°C. The supernatants were adjusted to 6 mM magnesium acetate, 200 µg/ml DNase I, 20 U/ml micrococcal nuclease and 1 mg/ml RNase A, and the reactions were incubated at 37°C for 45 min to digest nonencapsidated DNA. After adding EDTA to 10 mM final concentration, capsid-protected DNA was released by incubation for 3–5 h at 45°C with 800 µg/ml proteinase K and 5% SDS. Following phenol/chloroform extraction, HBV DNA was precipitated using 0.1 volumes of 10 M ammonium acetate, 60 µg/ml GlycogenBlue (Ambion) and 1 volume isopropanol, and washed using 70% ethanol. After gel electrophoresis, HBV specific DNAs were detected using a ^32^P-labeled probe obtained by random priming (NEBlot™ Kit) on a 3.2 kb EcoR I fragment containing a complete linear HBV genome.

### MMP8 and tMMP8 Detection by Immunoblotting

HepG2 cells were inoculated with Ad-CH-tMMP8, Ad-C-MMP8 and, for control, Ad-CH-RFP2. Four days later, immunoblotting after SDS-PAGE was performed as described [36] using a rabbit polyclonal MMP-8 antibody (Novus Biologicals, USA) which recognizes the segment (aa 107∼178) shared between MMP-8 and tMMP-8. Bands were visualized using a peroxidase-conjugated goat anti-rabbit IgG secondary antibody (ZSGB-Bio, China) and the enhanced chemiluminescence kit (Santa Cruz, USA). Tubulin on the same blot was detected as loading control.

### Rat Model of Liver Cirrhosis and Infection with Recombinant Adenoviruses

All animal experiments were approved by the Medical Ethics Committee of Bethune International Peace Hospital. Male Wistar rats were acquired from the Laboratory Animal Center of Hebei Medical University. To achieve cirrhosis, drinking water of the treatment group was adjusted to 0.03% (w/v) thioacetamide (TAA; Sigma-Aldrich) for 16 weeks [46]. Rats of the control group were reared in the same way but received normal tap water feeding. After validation of the cirrhosis model, the rats treated with TAA for 16 weeks were randomly divided into four experimental groups of 8 animals each. Ad virus particles were injected at 1.5×10^11^ VP/kg bodyweight through the tail vein.

### Detection of mRNAs for Full-length and Truncated MMP-8, Hepatocyte Growth Factor and c-Met

Total RNA from was extracted from HepG2 cells, or 100–150 mg rat liver tissue which was snap frozen and stored at −70°C prior to extraction, using TRIzol Reagent (Invitrogen) following the manufacturer’s instructions. Remaining DNA was removed by incubation with RNase-free Dnase I. First-strand cDNA was synthesized from 2 µg total RNA by SuperScript III reverse transcriptase (Invitrogen) with oligo(dT)_15_ primers as recommended by the manufacturer. Quantitative PCR (qPCR) was performed in an SLAN™ Real-Time PCR System (Hongshi, Shanghai, China) using the primers specific for MMP-8, tMMP8, hepatocyte growth factor (HGF; also known as scatter factor), c-Met, and glyceraldehyde-3-phosphate dehydrogenase (GAPDH) as listed in Table 1. Thermal cycling conditions comprised a predenaturation step at 94°C for 5 min and 45 cycles for amplification (denaturation at 94°C for 30 s, annealing at 52∼54°C for 30 s, extension and data collection at 72°C for 30 s). PCR was performed using the standard curve method for relative quantification of gene expression. Results for each sample were calculated as a ratio of the GAPDH concentration.

**Table 1:** PCR primers for MMP8, tMMP8, HGF, c-Met, and GAPDH mRNA.

Primers	Sequences	
tMMP-8	Forward	5-GGCGCCATGGTAACCCCAGGAAACC-3
	Reverse	5-GCCGACTAGTCATCCATAGATGGCCTGAATG-3
MMP-8	Forward	5-CCATCTATGGACTTTCAAGCAAC-3
	Reverse	5-TTGGAAGGGATGGCCAGAATAG-3
HGF	Forward	5-TCAAATGCCAGCCTTGGAATTCC-3
	Reverse	5-TCAAGAGTGTAGCACCATGGC-3
c-Met	Forward	5-AGCTGTACCTTGACCTTAAGC-3
	Reverse	5-TTCAGGGTCTTCCCAATACC-3
GAPDH	Forward	5-TCAAATGCCAGCCTTGGAATTCC-3
	Reverse	5-TCAAGAGTGTAGCACCATGGC-3

### Hepatic Hydroxyproline Determination

Liver samples of rats were obtained at the moment of sacrifice, and 150 mg of tissue were frozen, weighed and minced to homogeneity. Hepatic tissue (1 mg) was hydrolyzed with 2 ml 6N HCl for 12 h at 100°C. Hydroxyproline (HYP) content was determined by colorimetric assay at a wavelength of 560 nm as described [47]. The quantity of HYP was calculated against a calibration curve obtained using HYP standards (Sigma-Aldrich). Finally, the HYP content in each sample was normalized to the weight of liver tissue in which HYP was quantified.

### Sirius Red and Hematoxylin-eosin Staining

Liver tissues were fixed overnight in buffered formaldehyde (10%). After paraffin embedding, the liver tissues were sectioned at 5 µm in thickness. Sections were stained with 0.1% Sirius red in saturated picric acid to visualize collagen deposition [48] with a polarizing microscope. Area-density percent of collagen in fibrotic livers of rats was calculated using the Histogram module of Photoshop 8.0 software (Adobe). Hematoxylin–eosin (HE) staining was simultaneously performed to evaluate the degree of inflammation and pathological progression in liver with standard procedures. Modified Knodell scores for liver fibrosis [49] were determined to stage the inflammation and fibrosis of livers from rats of the different groups.

### Immunohistochemistry for Type I Collagen, MMP-8 and tMMP-8

Paraffin-embedded rat liver tissue sections were deparaffinized in xylene, rehydrated in alcohol, and incubated in 3% H_2_O_2_ to block endogenous peroxidase activity. Each section was incubated with normal goat serum for 30 min at room temperature to block nonspecific antibody-binding sites, then the slides were incubated at 4°C overnight with 1∶200 diluted mouse anti rat type I collagen antibody (Abcam), or 1∶200 diluted rabbit polyclonal antibody against human MMP-8 aa 107–178, respectively. After subsequent incubation with biotinylated goat anti-mouse IgG (Abcam) for 30 min at 37°C, each slide was twice rinsed in PBS. Peroxidase labeled streptavidin was applied and incubated for 20 min at room temperature according to manufacturer’s protocol. Sections were counterstained with hematoxylin (Sigma-Aldrich) for 1 min, then dehydrated and mounted.

### Statistical Analysis

All data are presented as mean ± standard deviation unless otherwise stated. Statistical analysis for parametric data was performed using Student’s t-test or, where appropriate, ANOVA, and for non-parametric data using Mann–Whitney’s U test. Differences were considered statistically significant at P values<0.05. All analyses were processed by SPSS for Windows ver 13.0 (SPSS, Chicago, IL, USA).

## Results

### Construction of HBV Vectors

The plasmid for the first transgene encoding HBV vector, pCH-S-GFP [28], was derived from the CMV promoter driven wild-type HBV expression vector pCH-9/3091 (Fig. 1A; [40]) by replacing the S gene with a gene for GFP such that GFP encoding mRNAs could be produced from the endogenous HBV sp1 and sp2 promoters normally controlling transcription of the 2.4 kb L protein (PreS1/PreS2/S) mRNA and the 2.1 kb M (PreS2/S) and S protein mRNAs [50]. Here we first generated additional analogous vectors encoding RFP2 and Renilla luciferase, termed pCH-S-RFP and pCH-S-Luc, respectively. A potential drawback of these vectors is that they can still express core protein and HBx protein, as well as C terminally truncated forms of the polymerase and L and M protein which may evoke undesired immune responses or act as transcriptional transactivators [51]. Similar to but not identical with (see below) a previous study [34] we therefore generated a second set of vectors that do not express endogenous HBV gene products. This was achieved by introducing artificial stop codons shortly after the initiation codons of the core, polymerase and X ORFs. For the preS1 and preS2 ORFs, rather than introducing premature stops codons [34], we mutated the initiation codons (Fig. 1). In this way, the transgene initiation codon becomes the first ATG also on the longer PreS2/S and PreS1 promoter driven RNAs which could then contribute to transgene expression (see below). The corresponding plasmids were termed pCH-M5, followed by the name of the encoded transgene. The pCH-S and pCH-M5 plasmids were later on used to generate the respective chimeric Ad-HBV vectors (Fig. 1).

**Figure 2:**
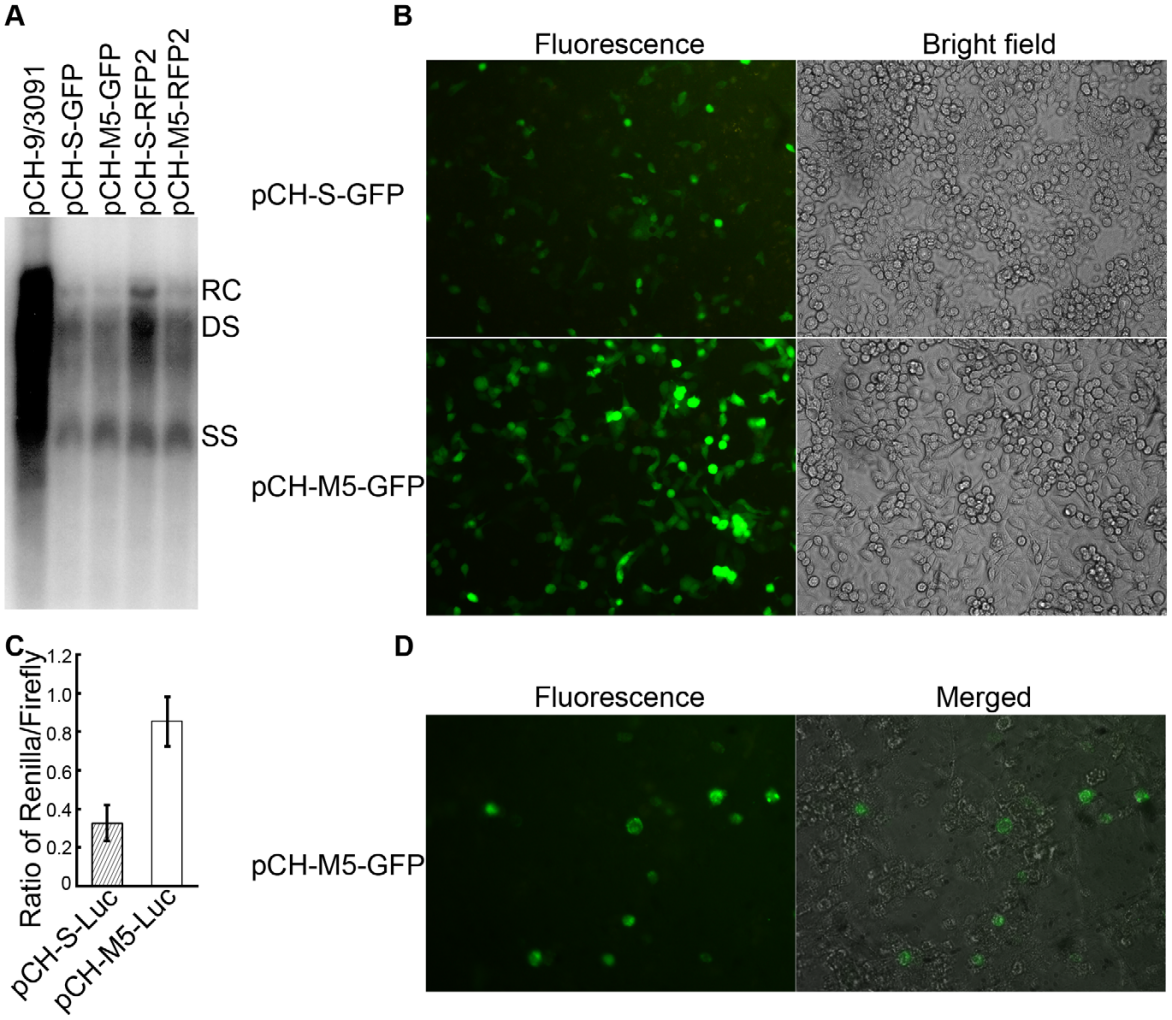
Trans-complementation rescues HBV vectors lacking endogenous gene products into infectious virions with enhanced transgene expression. (**A**) Rescue of replication in intracellular nucleocapsids by helper plasmid pCH3142. HepG2 cells were cotransfected with equal amounts of pCH3142 and the indicated pCH-S or pCH-M5 plasmids. Transfection with the wild-type HBV expression plasmid pCH-9/3091 served as reference. DNA from cytoplasmic nucleocapsids was analyzed by Southern blotting using a ^32^P-labeled HBV-specific probe. The positions of the major replicative intermediates, i.e. relaxed circular (RC) DNA, double-stranded linear DNA (DS) and single-stranded DNA (SS) are indicated. (**B**) **and (C) Enhanced transgene expression by HBV vectors devoid of endogenous gene products.** HepG2 cells were transfected with either the first generation pCH-S-GFP plasmid, or the new pCH-M5-GFP vector and GFP expression at 48 h post transfection was monitored by fluorescence microscopy (B). Alternatively, HepG2 cells were cotransfected with the Renilla luciferase encoding plasmids pCH-S-Luc or pCH-M5-Luc plus a plasmid encoding firefly luciferase (pGL3-control). At 48 h post transfection, Renilla luciferase activity from the HBV vector was then normalized to firefly luciferase activity in the same cells (C). (**D**) **Rescue of infectious HBV vector particle formation by wild-type HBV.** HepG2 cells were cotransfected with the vector pCH-M5-GFP and, instead of the helper plasmid pCH3142 as in (A), with the wild-type HBV expression plasmid pCH-9/3091. Viral particles from the supernatant were then tested for infectivity on differentiated HepaRG cells, using a nominal moi of 5,000 vge/cell. GFP expression indicating the presence of infectious vector particles was assessed by fluorescence microscopy 6 days post infection (panel “Fluorescence”). An overlay with the brightfield image of the same field is shown in panel “Merged”.

To assess the ability of the new vectors to be trans-complemented, the GFP and RFP2 encoding pCH-S and pCH-M5 vectors were co-transfected into HepG2 cells with the helper plasmid pCH3142 at a 1∶1 molar ratio. Previous data had already shown that on their own the pCH-S vectors (due to the massive alterations of the polymerase gene) and the helper plasmid (due to the deletion of the encapsidation signal) are unable to replicate [28], [30]. Cells transfected with the wild-type HBV vector pCH-9/3091 served as reference. Subsequently, DNA from intracellular nucleocapsids was analyzed by Southern blotting. As shown in Fig. 2A, trans-complemented pCH-S-GFP generated the typical DNA replication intermediates, i.e. relaxed circular (RC), double-strand linear (DS) and single-stranded (ss) DNA. The lower signal intensities compared to pCH-9/3091 transfected cells likely results from the preferential *cis*-packaging of the pgRNA from which polymerase is translated [52]. Essentially the same DNA patterns and similar signal intensities were produced by co-transfection of pCH3142 with the new pCH-M5 vectors. Hence neither replacement of the eGFP gene by the gene for RFP2 nor the mutations causing the knock-out of the endogenous HBV genes had a major impact on encapsidation or reverse transcription of the recombinant pgRNAs. Altogether, the modified vectors were as efficiently *trans*-complemented as the original pCH-S-GFP vector.

### Enhanced Transgene Expression by Abolishing the preS1 and preS2 Initiation Codons

Knocking out production of a functional gene product by premature termination will, in most cases, not allow translation initiation at downstream ATGs on the RNA and may even promoted nonsense-mediated mRNA decay [53]. Mutation of the preS1 and preS2 start codons should, in contrast, allow transgene expression from all preS1 and preS2/S promoter transcripts and may thus enhance transgene expression. To test this assumption, we transfected the fluorescent protein encoding pCH-S versus pCH-M5 vectors in the absence of helper plasmid into HepG2 cells and monitored the cells by fluorescence microscopy. As shown in Fig. 2B for the eGFP expressing vectors, the pCH-M5 vector caused a visibly stronger fluorescence. For quantitative assessment, we cotransfected HepG2 cells with a firefly luciferase expression plasmid (pGL3) plus either the pCH-S or pCH-M5 vector encoding Renilla luciferase. When normalized to the firefly luciferase control, Renilla luciferase activity was about 2.6-fold higher in pCH-M5-Luc than in pCH-S-Luc transfected cells (Fig. 2C); the difference was statistically significant (p<0.05). While other explanations are not excluded, these data were consistent with the intended increase in transgene expression level.

### Wild-type HBV can Support Formation of Infectious Recombinant Hepatitis B Virions *in trans*

Next we addressed whether the endogenous gene knockout HBV vectors could be rescued into infectious virions by wild-type HBV. To this end, we cotransfected HepG2 cells with equal amounts of the vector pCH-M5-GFPand the wild-type HBV plasmid pCH-9/3091; the GFP vector was chosen to allow direct detection of even a low percentage of infected cells. Successful *trans*-complementation should yield a mixture of wild-type and recombinant virions. Next we enriched viral particles from the culture supernatants and tested their infectivity in an *in vitro* infection system that is strictly dependent on an intact envelope, namely differentiated HepaRG cells [39]. About 10^5^ predifferentiated HepaRG cells per well of a six-well plate were incubated with about 5×10^8^ vge per well; this corresponds, combined for wild-type and potential recombinant virions, to a nominal multiplicity of infection (moi) of 5,000. As shown in Fig. 2D, in the range of 1% to 3% of the cells developed easily detectable green fluorescence which peaked around day 6 to day 8 post inoculation. Though seemingly low, this infection efficiency is not much below that reported for HepaRG cell infection with wild-type HBV which likely accounted for the majority of virions in the inoculum. For instance, at moi 1.25×10^4^ around 1% and even at moi 40×10^4^ not more than 7% of the cells became infected [54]. Similarly, others reported that at most 20% of the cells could be infected regardless of the amount of virions used [55]. While we have not directly tested the percentage of exclusively wild-type virus infected cells in our experiments, an additional factor may be the lack of intact HBx expression from the pCH-M5-GFP vector. Though not required for cccDNA formation, HBx appears to strongly promote transcriptional activity of the cccDNA [56]. This also implies that HBx was provided by co-infecting wild-type virus and/or that HBx dependence of cccDNA transcription is not absolute, as also suggested by previous data [34]. Also, other host factors might be crucial for cccDNA formation [57]. Clearly, however, GFP expression demonstrated that *cis*-preferential encapsidation of the wild-type pgRNA [52] did not abolish packaging and reverse transcription of the recombinant pgRNA, that the recombinant nucleocapsids received an intact envelope, and that the vector particles generated transcriptionally active cccDNA, all at levels allowing to easily detect recombinant virus infected cells. Infection-independent GFP expression from internalized vector plasmid is extremely unlikely (see Discussion).

**Figure 3:**
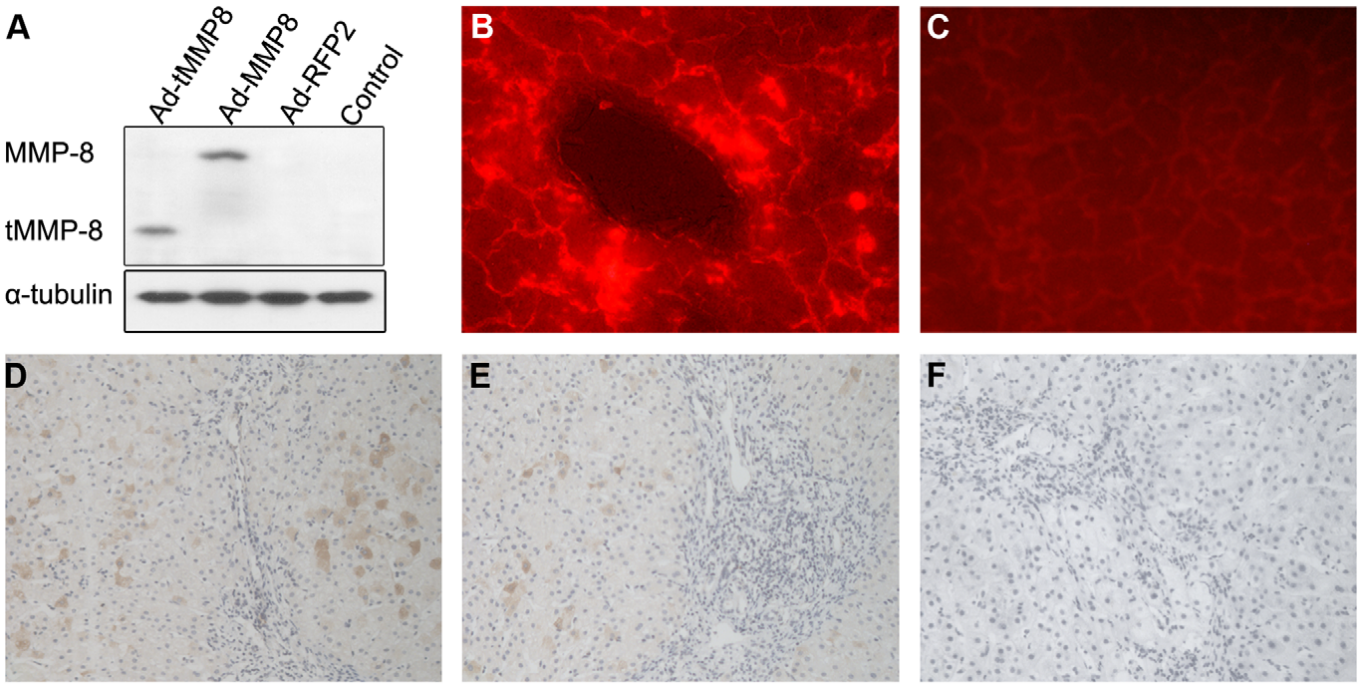
*In vitro* and *in vivo* transgene expression by chimeric Ad-HBV vectors. (**A**) **tMMP8 expression in HepG2 cells.** HepG2 cells were inoculated with the chimeric virus particles Ad-CH-tMMP8 (Ad-tMMP8) and Ad-CH-RFP2 (Ad-RFP2), or the conventional Ad-C-MMP8 particles; untreated HepG2 cells served as control. Four days later, cell lysates were analyzed by immunoblotting using a polyclonal MMP-8 antibody that recognizes an amino acid segment present in both MMP-8 and tMMP-8. Detection of tubulin on the same blot served as loading control. (**B**) and (**C**) Ad-HBV vector encoded transgene delivery into the liver. Ad-CH-RFP2 particles were injected into the tail vein of normal rats, with rats injected with saline as control. Two weeks later, liver tissues were immediately freeze-sectioned and analyzed for RFP2 expression by fluorescence microscopy. Strong red fluorescence was detected in rats treated with the Ad-HBV vector (B) whereas only limited autofluorescence was seen in rats treated with saline (C). (**D**)**,** (**E**) and (**F**) Rats with liver fibrosis were treated with Ad-CH-tMMP8 (D), Ad-C-MMP8 (E), or Ad-CH-RFP2 (F), respectively. Two weeks later, rat livers were sectioned and immunohistochemically stained with MMP-8 antibody. MMP-8 and tMMP-8 were visualized as brown precipitates.

Based on these data, we next generated an analogous vector for tMMP8, termed pCH-M5-tMMP8. With about 500 bp in length, the tMMP8 transgene is even smaller than the genes for eGFP (about 750 bp) and IFN-α (about 600 bp) that previously have) successfully been transduced using HBV vectors [28]. Subsequently, we used the pCH-M5 vectors for eGFP, RFP2, and tMMP8 to construct chimeric E1/E3-deleted (ΔE1/ΔE3) Ad-HBV vectors (Fig. 1), all of which were termed Ad-CH followed by the name of the HBV-embedded trans-gene. For reference, we generated a conventional Ad vector, Ad-C-MMP8, which encodes full-length MMP-8 under CMV promoter control (Fig. 1), as previously described [33]. To validate transgene expression by the Ad-HBV vectors, HepG2 cells were inoculated with Ad-CH-tMMP8, Ad-C-MMP8 or Ad-CH-RFP2; untreatedHepG2 cells served as negative control. Immunoblotting demonstrated that tMMP8 from the chimeric Ad-HBV vector was expressed at comparable levels as MMP8 from the conventional Ad vector (Fig. 3A). Successful liver transduction of the chimeric Ad-HBV vector encoded transgene, upon tail vein injection, was demonstrated by the development of strong fluorescence in liver sections from the RFP2 vector injected compared to non-injected rats (Fig. 3B and C).

### Rat Model of Liver Cirrhosis and Treatment Set-up

Long-term oral administration of 0.03% TAA in drinking water generates a well-established rat model of liver fibrosis and cirrhosis [20]. To confirm cirrhosis development in our setting, two animals each from the TAA treatment group were sacrificed at 4, 8, 12 and 16 weeks after initiation of treatment and their livers were tested by hematoxylin-eosin (HE) staining. Severe cirrhosis was confirmed in both animals sacrificed at the 16 week time point. Thirty-two of the remaining rats were then randomly divided into 4 groups (A, B, C and D) of 8 animals each. Rats of group A, B, and C were injected with 1.5×10^11^ virus particles (VP)/kg bodyweight of Ad-CH-tMMP8, Ad-C-MMP8, or Ad-CH-RFP2, respectively, through the tail vein. Animals from group D received physiological saline by the same route. The normal control group E consisted of eight weight-matched rats that had received normal drinking water without TAA.

To confirm expression of MMP8 and tMMP8, respectively, in fibrotic liver, liver tissue sections from TAA-treated rats of group A, B and C were immunohistologically evaluated with a polyclonal MMP-8 antibody recognizing an amino acid segment of MMP-8 that is also present in tMMP-8. Two weeks post virus injection, comparable cytoplasmic MMP-8 staining was seen in the livers of rats injected with Ad-C-MMP8 or Ad-CH-tMMP8 but not those having received Ad-CH-RPF2. (Fig. 3D, E and F). These data suggested the practical applicability of the chimeric Ad-HBV vectors in fibrotic livers.

Because the cirrhotic phenotype is reportedly maintained for at least 2 months after termination of TAA feeding [21], [58], rats were sacrificed 2, 4, 8 weeks after Ad vector or saline injection to evaluate potential therapeutic effects. Fibrosis was assessed by determining standard parameters, including hepatic hydroxyproline content, morphological and histological changes, Sirius red staining, and Knodell histologic activity index as modified by Wang et al. [49], [59].

### Similar Reduction in Elevated Hydroxyproline Content of Cirrhotic Livers by Ad-C-tMMP8 and Ad-CH-tMMP8 Treatment

**Figure 4:**
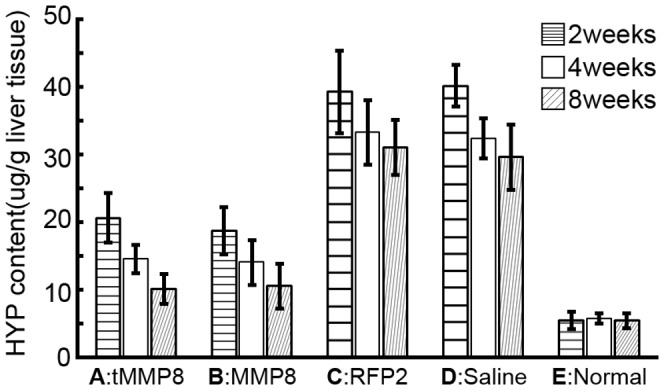
Chimeric Ad-HBV vector delivering truncated MMP-8 achieves similar reduction of hydroxyproline content in cirrhotic liver as a conventional full-length MMP-8 Ad vector. Hydroxyproline (HYP) contents in livers from normal rats (group E), or rats with TAA-induced cirrhosis (groups A–D) and treated with the chimeric Ad-HBV vectors Ad-CH-tMMP8 (group A) or Ad-CH-RFP2 (group C), or the conventional Ad vector Ad-C-MMP8 (group B) or saline (group D) were determined 2 weeks, 4 weeks, and 8 weeks after treatment, using a standard colorimetric assay. Both MMP-8 vectors achieved a significant reduction in HYP content compared to the Ad-CH-RFP2 vector and saline. Error bars represent standard deviation (n = 3).

Hydroxylation of prolines stabilizes collagen, and increased HYP content is a marker for fibrosis. As shown in Fig. 4, the HYP contents in the control group E remained constant over time at around 5–7 µg/g liver whereas they were significantly higher in all TAA treated rats, with a gradual decline from week 2 to week 8 after Ad vector or saline administration (Fig. 4). The highest values (around 40 µg/g) were found in the animals that had received the Ad-CH-RFP2 vector or saline (p<0.05 compared to the normal group at all time points). The lack of significant difference between these two groups showed that the Ad vector as such had no measurable effect. Much in contrast, HYP contents in the rats injected with either Ad-CH-tMMP8 or Ad-C-MMP8 were two-fold lower already at two weeks after vector administration, and around three-fold lower at the 8 week timepoint (p<0.05 at all three time points). Notably, with about 10 µg/g the levels at week 8 were only slightly higher than in the normal control compared to still around 30 µg/g in the Ad-CH-RFP2 and saline injected rats (p<0.05 compared to the normal controls). Hence the conventional full-length MMP-8 Ad vector and the chimeric Ad-HBV vector for truncated tMMP-8 were similarly effective in reducing the fibrosis marker HYP.

### Similar Amelioration of Cirrhotic Phenotype by Ad-C-MMP8 and Ad-CH-tMMP8 Vector Administration

**Figure 5:**
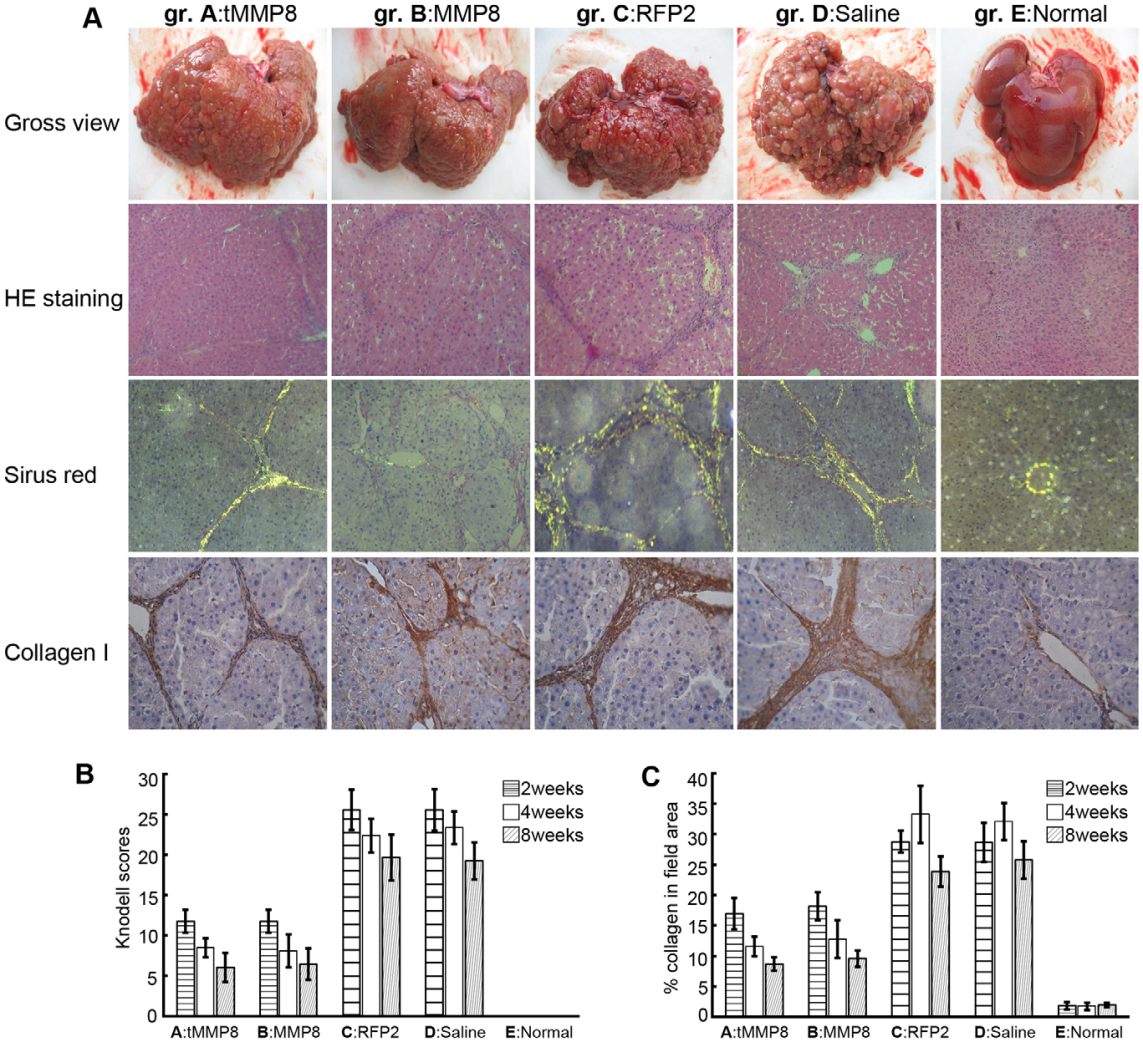
Chimeric Ad-HBV vector delivering truncated MMP-8 achieves similar amelioration of fibrosis and cirrhosis as a conventional full-length MMP-8 Ad vector. (**A**) Gross morphology and histology. Livers and liver sections from rats of the same treatment groups as in Fig. 4 were analyzed for gross morphology (Gross view), and by hematoxylin - eosin (HE) staining, Sirius red collogen staining, or immunohistochemical staining specific for type I collagen. (**B**) Overall collagen contents. Sirius red staining followed by polarizing microscopy was used to determine the collagen positive areas per liver sections. (**C**) Knodell fibrosis scores. The highly elevated collogen levels and Knodell scores in the cirrhotic animals treated with the RFP2 Ad vector or saline were strongly reduced by treatment with either the full-length MMP-8 Ad vector or the chimeric Ad-HBV vector delivering truncated tMMP8. Error bars represent standard deviations (n = 3).

Visual inspection of the gross liver morphologies eight weeks after Ad vector or saline administration (Fig. 5A, panel Gross view) revealed massive changes compared to the normal control, particularly for the TAA treated rats that had received the Ad-CH-RFP2 vector or saline. While the normal livers exhibited ordinary reddish-rufous color, perfectly smooth surface, and regular shape, livers from the latter two groups showed more or less shrinkage, abnormal color, irregular outlines, and a strikingly uneven surface with protruding mixed-sized fibrotic nodules. Histology confirmed numerous regenerative parenchyma nodules surrounded by septa of fibrous tissue, and a significant increase in fat storing cells, Kupffer cells and bile ductules. In contrast, livers of rats injected with Ad-CH-tMMP8 or Ad-C-MMP8 presented with much milder patho-morphological progression, with fewer and smaller fibrotic nodules on the liver surface and softer hepatic parenchyma. HE-staining eight weeks after Ad-CH-tMMP8 or Ad-C-MMP8 administration (Fig. 5A, panel HE staining) showed a relatively normal aspect and subcellular structure of hepatocytes, with well-preserved cytoplasm and prominent nuclei and nucleoli. Appreciable histological regeneration was indicated by a reduced extent of fibrous septa and an increase in the extension of normal hepatic parenchyma. Knodell scores of livers from rats injected with Ad-CH-tMMP8 or Ad-C-MMP8 were evidently lower than in rats treated by Ad-CH-RFP2 or saline (Fig. 5B), and the differences were statistically significant at all time points (p<0.05). No significant histological differences were observed between Ad-CH-tMMP8 and Ad-C-MMP8 injected animals. Sirius red staining eight weeks after Ad vector or saline administration followed by polarized light microscopy can be used to visualize the collagen in fibrotic or cirrhotic livers (Fig. 5A, panel Sirius red), and to semi-quantitatively calculate the collagen content by area-density percentage of collagen in liver sections [48]. As shown in Fig. 5C, compared to the normal control these values were strongly increased (around 30%) in livers of TAA treated animals that had received Ad-CH-RFP2 or saline, indicating severe destruction of hepatic lobules and frequent pseudolobuli formation accompanied by impaired hepatic function. In contrast, significantly (p<0.05) lower values (around 17% at week 2, declining to around 10% at week 8) were observed in the groups that had received Ad-C-MMP8 and Ad-CH-tMMP8. These data further supported that tMMP8 embedded in the Ad-HBV vector was similarly efficient in reducing pathological collagen deposition as the conventional full-length MMP8 Ad vector.

This was further confirmed by immunocytochemistry using a type I collagen-specific antibody eight weeks after Ad vector or saline administration (Fig. 5A, panel Collagen I). Livers from the control group E showed only weak brownish staining that was sporadically distributed around vessel walls. In all TAA treated animals, abundant positively staining structures accumulated in portal areas, portal veins, fibrous septa and hepatic stellate cells. In contrast, type I collagen congestion in rats injected with Ad-CH-tMMP8 and Ad-C-MMP8 was markedly reduced when compared to the RFP2 vector and saline injected controls, and further decreased gradually from week 2 to week 8 after treatment. Furthermore, the therapeutic effect was extended up to 8 weeks after adenovirus infection (Fig. 5A, panel Collagen I).

### Similar Elevated Hepatocyte Growth Factor and c-Met mRNA Levels in Ad-C-MMP8 and Ad-CH-tMMP8 Transduced Livers

Removal of excess ECM not only frees physical space for hepatocytes but also acts negatively on activated HSC, the main ECM producers, and positively on hepatocyte proliferation [18], possibly by liberating ECM-bound HGF. Because Ad vector-mediated transduction of full-length MMP-1 and MMP-8 was reported to induce hepatocyte proliferation [21], [33] and HGF upregulation, we investigated whether truncated tMMP8 delivered by the chimeric Ad-HBV vector would have a similar effect. In addition, we included the HGF receptor c-Met [60] into the analysis, considering that activation of the HGF/c-Met signaling pathway is one of the earliest events towards hepatocyte regeneration following partial hepatectomy [60], [61], [62], [63].

**Figure 6:**
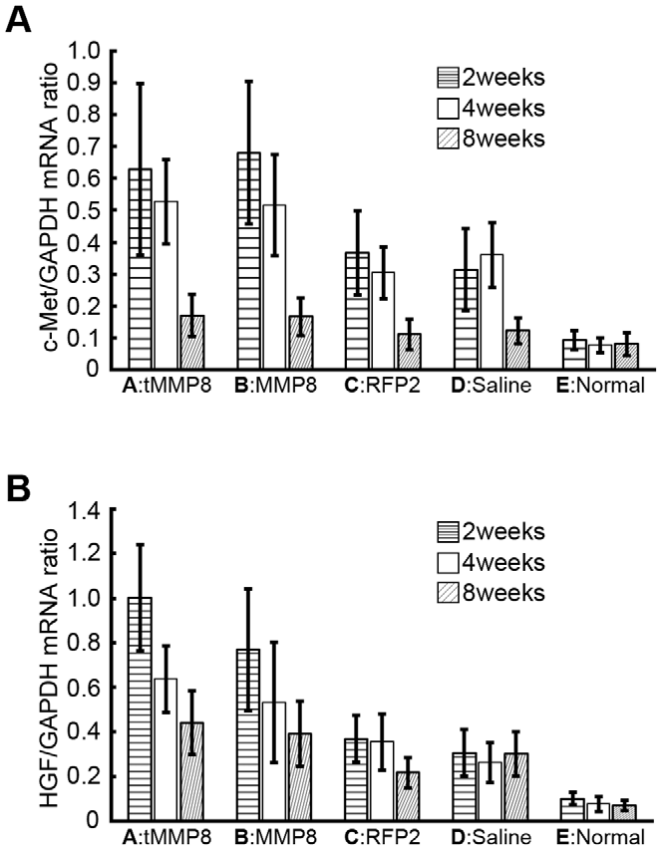
Similar enhancement of hepatocyte proliferation promoting factors by chimeric Ad-HBV vector for truncated MMP-8 and conventional full-length MMP-8 Ad vector. (**A**) c-Met mRNA. (**B**) Hepatocyte growth factor mRNA. mRNA levels for c-Met, hepatocyte growth factor (HGF) and glyceraldehyde 3-phosphate dehydrogenase (GAPDH) in liver tissue from the same treatment groups as in Fig. 3 were determined by quantitative RT-PCR at the indicated timepoints post treatment. Values for c-Met and HGF were then correlated with those for GAPDH in the same sample. Error bars represent standard deviations (n = 3).

To this end, we used qPCR to determine the levels of c-Met and HGF mRNA relative to the levels of the mRNA for the house-keeping gene product GAPDH. Compared to the normal control, relative mRNA levels for both HGF and c-Met were slightly elevated in the TAA cirrhotic animals injected with Ad-CH-RFP2 or saline, but were markedly higher in the rats that had received the Ad-C-MMP8 and the Ad-CH-tMMP8 vector, especially at the early timepoints after administration (Fig. 6A and B). The differences to the Ad-CH-RFP2 and saline controls were significant (p<0.05), while those between the two MMP-8 vectors were not (Fig. 6A and B, Group A,B vs Group C,D, p<0.05). Upregulation of HGF and c-Met mRNA could be detected as long as 8 weeks after injection of the MMP-8 vectors, implying a similarly sustained hepatocyte proliferation promoting effect by the chimeric Ad-HBV vector encoding truncated tMMP8 as by the conventional Ad-vector encoding full-length MMP-8.

## Discussion

Liver fibrosis prepares the ground for cirrhosis and, especially in the context of chronic hepatitis B, may directly progress into hepatocellular carcinoma in some 40% of the patients [2]. Removing the injurious stimuli underlying liver fibrosis appears to promote fibrosis regression and possibly even reversion [2] but treatment of advanced fibrosis and cirrhosis probably require more direct means of ECM remodeling. Transduction of MMP genes by Ad vectors has shown promise [18], [21], [33] yet application of conventional Ad vectors in humans has met with several concerns [23], particularly when high doses of vector are required to reach a sufficient fraction of liver cells without affecting other tissues.

The previous demonstration that the naturally hepatotropic HBV can be engineered into a liver-specific gene transfer vehicle [28], [34] suggests HBV-based vectors as a potential alternative in the treatment of liver diseases. A general restriction is their limited insertion capacity, requiring finding a suitably sized effector gene. A second, relevant for HBV-related diseases, is superinfection exclusion. Though not absolute, as shown experimentally [28] and by the frequent takeover in chronic HBV carriers by resistant virus variants during treatment with early generation nucleoside analogs [64], resident HBV might substantially reduce transduction by HBV-based vectors, as shown for DHBV [32]. Conversely, once in an HBV-infected cell, a replication-defective HBV vector might be amplified and spread until the supply of wild-type virus ceases, e.g. by antiviral treatment. Thus, an appropriately designed HBV vector treatment might achieve a longer term expression of therapeutic genes than high-dose administration of conventional Ad-vectors, yet avoid the risks associated with permanently integrating retroviral or lentiviral vectors.

However, various prerequisites for seriously considering such advanced application regimens were unknown. These include whether *trans*-complementation by wild-type HBV rather than a purposefully engineered helper construct would occur to practically useful levels, whether a truncated MMP-8, sufficiently small to fit into an HBV vector, would exert similarly positive effects against fibrosis and cirrhosis as full-length MMP-8, and whether such an HBV-vector could be shuttled into liver cells and express the transgene with similar efficiency as a conventional Ad-vector. Such shuttling should provide the means for efficient initial deposition of the HBV vector despite superinfection exclusion.

The current study addresses, for the first time in an *in vivo* liver disease model, several of these questions. Given the lack of animal models that combine susceptibility to HBV infection with human-like disease progression, some aspects had to be assessed using surrogate models. Hence it remains to be shown whether vector complementation by resident wild-type HBV occurs *in vivo*, and whether potential vector spreading can occur in fibrotic/cirrhotic liver, an issue that is currently unclear even for wild-type HBV. Altogether, however, our other data strongly suggest that further investments into developing HBV-vectors for liver diseases including, but not limited to, fibrosis and cirrhosis are warranted.

### Improved HBV Vector Design and Trans-complementation by Wild-type HBV

The first generation HBV vectors retained the potential to generate core and HBx protein, as well as truncated forms of polymerase and L and M protein [28]. For application in HBV-infected cells anti-HBV vector immune responses would be of less concern, but undesired side-effects could arise from the reported transactivation activity of truncated M and L protein [65], [66]. Similar to a previous study [34], we therefore knocked out expression of all residual vector encoded viral gene products, including intact HBx (see below). However, different from that study we prevented expression of the preS1 and preS2 ORFs by mutating the respective start codons such that all transcripts from the preS1 and preS2/S promoters would be usable as mRNAs for the transgenes. Indeed, the pCH-M5-GFP vector produced visibly more GFP than the parental pCH-S-GFP vector, as was quantitatively confirmed using the corresponding luciferase constructs (Fig. 2B and 2C). In addition, the distinct hepatocyte specificity especially of the preS1 promoter [29] should contribute to liver-specific transgene expression. Alternatively, a previous study [34] as well as our own unpublished data indicate that transgene expression may be achieved by replacing the entire preS1, preS2 plus S regions with expression cassettes comprising a heterologous promoter, and that such cassettes are tolerated at a few other sites in the HBV genome. Such flexibility may turn out to be advantageous in further HBV vector improvement.

The knock out mutations in pCH-M5-GFP did not impair trans-complementation by the pCH3142 helper plasmid, as shown by the approximately equal amounts of replicative DNA intermediates obtained by cotransfection with the parental versus the new vectors (Fig. 2A). More relevant regarding potential vector amplification by wild-type HBV was the apparent formation of infectious, GFP-transducing vector particles by trans-complementation with the wild-type HBV vector pCH-9/3091, although only a low percentage of the inoculuated HepaRG cells became GFP-positive (Fig. 2D). Several factors are likely to contribute to this result. First, only a fraction of the HepaRG cells is susceptible even to wild-type HBV infection [54]. Second, given the choice, HBV polymerase packages the pgRNA from which it was translated at four- to ten-times higher efficiency than a simultaneously present pgRNA on which polymerase translation was ablated [52]; with the encapsdiation-deficient pCH3142 helper plasmid such competition for pgRNA packaging is deliberately excluded. Third, the pCH-M vector does not produce intact HBx which according to recent results with wild-type HBV infected HepaRG cells is not required for HBV cccDNA formation but strongly enhances its transcriptional activity [56]. One interpretation for visible GFP expression in our experiments is a low level HBx-independent cccDNA transcription, as previously seen with HBx-deficient vectors [28], [34] and also with wild-type HBV [56]. Another is that the GFP-positive cells had been co-infected with wild-type HBV which was likely present in the inoculum in substantial excess and provided HBx. While additional experiments will be required for distinction, the data strongly suggest that vector *trans*-complementation by wild-type HBV is principally possible and comprises all processes required for infectious virion formation. A trivial explanation would be GFP expression from internalized pCH-M-GFP plasmid; however, we consider this extremely unlikely. First, the total amount of viral DNA inoculated was in the nanogram range (around 5×10^8^ vge per well) whereas microgram amounts plus an efficient transfection reagent are required to achieve expression of a plasmid-encoded gene in a substantial fraction cells (in the range of 20% for HepG2 cells which probably more easily transfected than HepaRG cells). Second, free DNAs not protected inside viral particles had been digested by DNase I and micrococcal nuclease prior to enrichment of the virions used for inoculation (see Materials and Methods).

Definite proof for vector *trans*-complementation by wild-type HBV *in vivo* would require an appropriate animal infection model such as uPA-SCID mice xenotransplanted with human or tupaia hepatocytes [67]. However, this model is highly complex and not readily available, and as yet no fibrosis or cirrhosis model based on this infection system has been reported.

### Similar Therapeutic Benefit of an Ad-vector Shuttled HBV Vector Encoding TruncaTed tMMP8 as of a Conventional Ad-vector Encoding Full-length MMP-8

The previously observed benefits of full-length MMP-1 and MMP-8 delivery into rat models of liver cirrhosis by conventional Ad-vectors [21], [33] provided a standard against which the performance of our Ad-vector-shuttled HBV tMMP8 vector could be compared. Initial experiments confirmed about equal expression of tMMP8 from the Ad-HBV vector versus MMP8 from the conventional Ad vector in infected HepG2 cells (Fig. 3A), and productive *in vivo* delivery of an Ad-HBV vector encoded reporter transgene into the liver of normal rats by tail-vein injection (Fig. 3B,C). Confirming earlier studies [20], 16 week administration of TAA induced severe fibrosis and cirrhosis in all rats tested, as shown by gross morphological and histological comparison with normal rat liver (Fig. 4 and Fig. 5). In particular, various qualitative and quantitative parameters reflecting the hallmark of fibrosis, i.e. excess interstitial collagen-containing ECM deposition, were strongly elevated in the TAA treated animals. Notably, in all assays, including HYP content (Fig. 4), area-density percentage of collagen (Fig. 5B) or Knodell score (Fig. 5C), the cirrhotic animals injected with the Ad-CH-RFP2 vector or saline showed significantly higher elevations than the Ad-C-MMP8 and Ad-CH-tMMP8 treated animals, correlating with detectable MMP8 expression in the latter but not the former groups (Fig. 6 D, E vs. F). In contrast, no significant differences were seen amongst the two control groups and amongst the two MMP-8 vector treated groups. The lack of difference between Ad-CH-RFP2 vs. saline injected rats implies that their assay parameters were representative for the degree of TAA-induced fibrosis in all animals of the cohort, and it further confirmed that the Ad-CH vector *per se* had no detectable influence. The equal reduction in fibrotic and cirrhotic parameters in the tMMP-8 versus MMP8 vector-treated groups indicated that both caused a significant and comparable therapeutic benefit compared to the RFP2 vector or saline controls. This may suggest that vector amplification by resident HBV, not testable in this rat model, would even further enhance therapeutic efficacy of the HBV vector yet proof will require a disease model that is susceptible to HBV infection.

Notably, however, in another, testable, aspect the tMMP8 chimeric Ad-HBV vector proved again as effective as the conventional full-length MMP8 Ad vector. In the previous studies on MMP-1 and MMP-8 transduction the induction of hepatocyte proliferation [38] was noted as an additional benefit accompanying fibrosis regression. Though various mechanisms may account for this effect, the ECM itself probably plays an important part. Excessive ECM further stimulates activated HSCs, whereas ECM reduction favors hepatocyte proliferation, possibly by liberating ECM-bound HGF [18]. HGF signaling through its receptor kinase c-Met [60] is indeed the major initial stimulus for liver regeneration after partial hepatectomy [61], [62], [63]. Our corresponding qPCR analysis of HGF and c-Met mRNA (Fig. 6) revealed significantly (compared to the normal control) and about equally elevated levels of both mRNAs in the animals receiving the two different MMP-8 vectors. Smaller increases, compared to the normal control, were also observed in the cirrhotic animals injected with the RFP2 vector or saline, which may reflect spontaneous liver regeneration after cessation of the TAA treatment. Importantly, the lack of difference between the RFP2 and saline controls further confirmed that the elevated HGF and c-Met mRNA levels can be attributed to the vector-encoded MMP-8 genes.

### Implications for Future Application of HBV-based Vectors Against Liver Diseases

Although efficient and safe delivery of therapeutic genes remains challenging, with none of the currently used viral and non-viral systems being ideal, HBV-based vectors offer distinct conceptual advantages for liver-specific gene therapy [31]. A unique feature is their potential for limited amplification by exploiting resident wild-type virus as a natural helper; however, this potential may be counteracted by superinfection exclusion. Our study demonstrates that HBV vectors can exert therapeutic benefit in an *in vivo* model of liver fibrosis and cirrhosis, which are relevant liver diseases often associated with HBV infection; that HBV vectors can be complemented by wild-type HBV; and that an unrelated vector that is not subject to HBV-specific superinfection exclusion can efficiently shuttle an HBV vectors into the liver. Hence further development of this or similar strategies for efficient initial deposition of an HBV vector by a low, and consequently low-risk, dose of appropriate shuttling vectors appears highly warranted.
